# Tetra­hedral zinc in tetra­kis­(1-methyl-1*H*-imidazole-κ*N* ^3^)zinc bis­(tetra­fluorido­borate)

**DOI:** 10.1107/S1600536811054821

**Published:** 2011-12-23

**Authors:** Jan Reedijk, Gerard A. van Albada, Bart Limburg, Ilpo Mutikainen, Urho Turpeinen

**Affiliations:** a Leiden Institute of Chemistry, Leiden University, PO Box 9502, 2300 RA Leiden, The Netherlands; b Department of Chemistry, College of Science, King Saud University, PO Box 2455 Riyadh 11451, Kingdom of Saudi Arabia; c University of Helsinki, Department of Chemistry, Laboratory of Inorganic Chemistry, FIN-00014 Helsinki, Finland

## Abstract

In the title compound, [Zn(C_4_H_6_N_2_)_4_](BF_4_)_2_, the Zn^II^ ion is in a slightly distorted tetra­hedral coordination geometry, with Zn—N distances in the range 1.980 (2)–1.991 (2) Å. The tetra­hedral angles are in the range 104.93 (9)–118.81 (9)°.

## Related literature

For related structures, see: Chen *et al.* (1996 ▶). For the synthesis and properties of the title compound, see: Reedijk (1969 ▶). The crystal was mounted using the oil-drop method, see: Kottke & Stalke (1993 ▶).
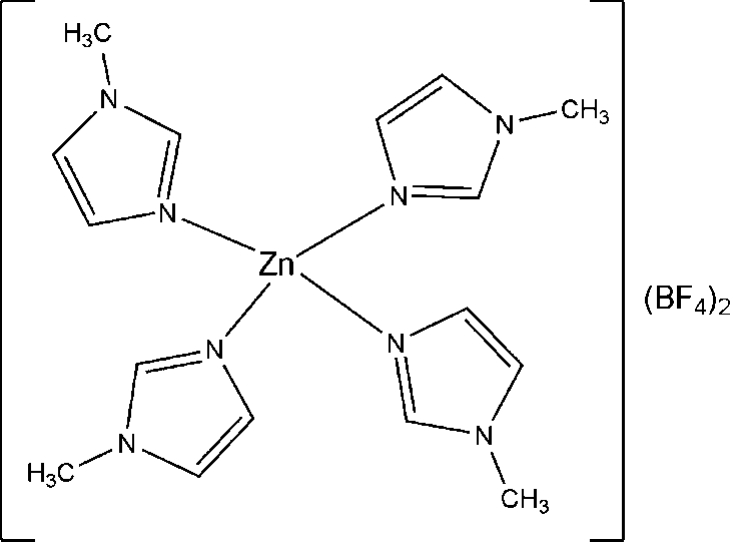


## Experimental

### 

#### Crystal data

[Zn(C_4_H_6_N_2_)_4_](BF_4_)_2_
*M* *_r_* = 567.42
Orthorhombic, 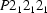

*a* = 7.257 (1) Å
*b* = 16.023 (1) Å
*c* = 21.040 (2) Å
*V* = 2446.5 (4) Å^3^
*Z* = 4
Mo *K*α radiation
μ = 1.09 mm^−1^
*T* = 173 K
0.30 × 0.30 × 0.20 mm

#### Data collection

Nonius KappaCCD diffractometer
Absorption correction: multi-scan (*SADABS*; Sheldrick, 1996) ▶ *T*_min_ = 0.737, *T*_max_ = 0.812
17949 measured reflections
4184 independent reflections
3782 reflections with *I* > 2σ(*I*)
*R*_int_ = 0.024

#### Refinement

*R*[*F* ^2^ > 2σ(*F* ^2^)] = 0.027
*wR*(*F* ^2^) = 0.068
*S* = 1.08
4184 reflections
320 parameters
H-atom parameters constrained
Δρ_max_ = 0.29 e Å^−3^
Δρ_min_ = −0.22 e Å^−3^
Absolute structure: Flack (1983 ▶), 1690 Friedel pairs
Flack parameter: 0.038 (11)

### 

Data collection: *COLLECT* (Nonius, 2002 ▶); cell refinement: *DIRAX* (Duisenberg, 1992 ▶); data reduction: *COLLECT*/*EVAL* (Nonius, 2002 ▶); program(s) used to solve structure: *SHELXS97* (Sheldrick, 2008 ▶); program(s) used to refine structure: *SHELXL97* (Sheldrick, 2008 ▶); molecular graphics: *SHELXTL* (Sheldrick, 2008 ▶); software used to prepare material for publication: *SHELXTL*.

## Supplementary Material

Crystal structure: contains datablock(s) I, global. DOI: 10.1107/S1600536811054821/bv2196sup1.cif

Structure factors: contains datablock(s) I. DOI: 10.1107/S1600536811054821/bv2196Isup2.hkl

Additional supplementary materials:  crystallographic information; 3D view; checkCIF report

## Figures and Tables

**Fig. 1.**
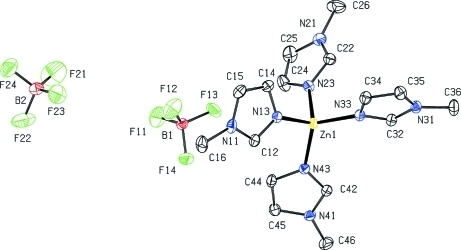
Showing the structure of tetrakis(N-methylimidazole)zinc(II) bis(tetrafluoridoborate) with atom labeling. Atomis displacement parameters at the 50% level. Hydrogen atoms omitted for clarity.

## supplementary crystallographic information

### Comment

The ligand 1-methyl-1*H*-methylimidazole (Meim) is an often used solvent and ligand for transition metal ions (Reedijk, 1969). It is a sterically non-demanding heterocyclic ligand, and it readily forms octahedrally coordinated homoleptic compounds with all first-row transition metal ions; the only exception is Cu(II), where 4 ligands coordinate together with 2 anions or other ligands, in a tetragonal geometry; this deviating behaviour is ascribed to the Jahn-Teller effect that prevents d^9^ ions from having high symmetry. Remarkably, and in addition to the six-coordinate Zn^II^ species, in the case of Zn also tetrahedrally coordinated homoleptic compounds were reported by one of us, for both perchlorate and tetrafluoridoborate (Reedijk, 1969). The tetrahedral geometry was deduced from the significantly different IR ring vibrations near 955 cm^-1^, compared to the octahedral cases (Reedijk, 1969). Proof for this structure was lacking and another structure, like a tetragonal case with 2 anions could not be excluded. Sometime ago a room temperature three-dimensional structure was reported for the perchlorate, albeit with a less high accuracy (Chen *et al.* 1996). We now report the related tetrafluoridoborate, which is not isomorphous with the perchlorate, in high accuracy. The molecular structure differs hardly from the perchlorate, and the Zn—N distances are slightly shorter, just as one would expect for the present low-temperature structure.

### Experimental

0.005 mol of hydrated zinc tetrafluoroborate, [Zn(H_2_O)_6_](BF_4_)_2_ is reacted in a 100 ml conical flask with 3 ml of trimethyl orthoformate, mof = (CH_3_O)_3_CH and the reaction mixture is dissolved in about 25 ml of methanol. Add to this metal salt solution a solution (*drop by drop !!*) of 0.01 mol of Meim in 10 ml of methanol. Crystals appear upon standing, and can be enhanced by slow evaporating of some of the solvent or after addition of some diethyl ether. The crystals were characterized by elemental analysis and infrared spectra and shown to be identical to the 1969 sample.

A crystal was selected for the X–ray measurements and mounted to the glass fiber using the oil drop method (Kottke & Stalke, 1993) and data were collected at 193 K. The intensity data were corrected for Lorentz and polarization effects and for absorption.

### Refinement

*DIRAX* Software was used for the unit cell refinement (Duisenberg 1992) *SHELXL97* was used for the structure refinement (Sheldrick, 2008). All hydrogen atoms were fixed geometrically and allowed to ride on the parent carbon atoms, with aromatic C—H = 0.93 Å, methyl C—H = 0.96 Å and methylene C—H = 0.97 Å. The displacement parameters were set for phenyl and methylene H atoms at *U*_iso_(H) = 1.2*U*_eq_(C) and methyl H atoms at *U*_iso_(H) = 1.5*U*_eq_(C).

### Crystal data

**Table:**

[Zn(C_4_H_6_N_2_)_4_](BF_4_)_2_	*F*(000) = 1152
*M**_r_* = 567.42	*D*_x_ = 1.541 Mg m^−^^3^
Orthorhombic, *P*2_1_2_1_2_1_	Mo *K*α radiation, λ = 0.71073 Å
Hall symbol: P 2ac 2ab	Cell parameters from 17949 reflections
*a* = 7.257 (1) Å	θ = 2–25°
*b* = 16.023 (1) Å	µ = 1.09 mm^−^^1^
*c* = 21.040 (2) Å	*T* = 173 K
*V* = 2446.5 (4) Å^3^	Block, colourless
*Z* = 4	0.30 × 0.30 × 0.20 mm

### Data collection

**Table:**

Nonius KappaCCD diffractometer	4184 independent reflections
Radiation source: fine-focus sealed tube	3782 reflections with *I* > 2σ(*I*)
graphite	*R*_int_ = 0.024
φ–scan	θ_max_ = 25.1°, θ_min_ = 2.5°
Absorption correction: multi-scan (*SADABS*; Sheldrick, 1996)	*h* = −8→8
*T*_min_ = 0.737, *T*_max_ = 0.812	*k* = −19→19
17949 measured reflections	*l* = −25→19

### Refinement

**Table:**

Refinement on *F*^2^	Secondary atom site location: difference Fourier map
Least-squares matrix: full	Hydrogen site location: inferred from neighbouring sites
*R*[*F*^2^ > 2σ(*F*^2^)] = 0.027	H-atom parameters constrained
*wR*(*F*^2^) = 0.068	*w* = 1/[σ^2^(*F*_o_^2^) + (0.0283*P*)^2^ + 0.9793*P*] where *P* = (*F*_o_^2^ + 2*F*_c_^2^)/3
*S* = 1.08	(Δ/σ)_max_ = 0.002
4184 reflections	Δρ_max_ = 0.29 e Å^−^^3^
320 parameters	Δρ_min_ = −0.22 e Å^−^^3^
0 restraints	Absolute structure: Flack (1983), 1690 Friedel pairs
Primary atom site location: structure-invariant direct methods	Flack parameter: 0.038 (11)

### Special details

**Table:**

Geometry. All e.s.d.'s (except the e.s.d. in the dihedral angle between two l.s. planes) are estimated using the full covariance matrix. The cell e.s.d.'s are taken into account individually in the estimation of e.s.d.'s in distances, angles and torsion angles; correlations between e.s.d.'s in cell parameters are only used when they are defined by crystal symmetry. An approximate (isotropic) treatment of cell e.s.d.'s is used for estimating e.s.d.'s involving l.s. planes.
Refinement. Refinement of *F*^2^ against ALL reflections. The weighted *R*-factor *wR* and goodness of fit *S* are based on *F*^2^, conventional *R*-factors *R* are based on *F*, with *F* set to zero for negative *F*^2^. The threshold expression of *F*^2^ > σ(*F*^2^) is used only for calculating *R*-factors(gt) *etc*. and is not relevant to the choice of reflections for refinement. *R*-factors based on *F*^2^ are statistically about twice as large as those based on *F*, and *R*- factors based on ALL data will be even larger.

### Fractional atomic coordinates and isotropic or equivalent isotropic displacement parameters (Å^2^)

**Table:**

	*x*	*y*	*z*	*U*_iso_*/*U*_eq_	
Zn1	0.74735 (4)	0.106549 (16)	0.211095 (12)	0.02792 (9)	
N11	1.0279 (3)	0.18745 (14)	0.36966 (11)	0.0349 (5)	
C12	0.9127 (4)	0.13756 (17)	0.33848 (13)	0.0333 (6)	
H12A	0.8408	0.0951	0.3579	0.040*	
N13	0.9111 (3)	0.15463 (13)	0.27699 (10)	0.0293 (5)	
C14	1.0326 (4)	0.21893 (16)	0.26899 (14)	0.0331 (6)	
H14A	1.0620	0.2448	0.2297	0.040*	
C15	1.1040 (4)	0.23962 (18)	0.32598 (15)	0.0380 (7)	
H15A	1.1911	0.2826	0.3341	0.046*	
C16	1.0587 (5)	0.1890 (2)	0.43885 (14)	0.0521 (9)	
H16A	1.0325	0.1337	0.4566	0.078*	
H16B	0.9769	0.2303	0.4584	0.078*	
H16C	1.1872	0.2038	0.4476	0.078*	
N21	0.5616 (4)	0.28245 (15)	0.08385 (11)	0.0407 (6)	
C22	0.6664 (4)	0.22009 (18)	0.10313 (14)	0.0389 (7)	
H22A	0.7591	0.1946	0.0778	0.047*	
N23	0.6270 (3)	0.19746 (14)	0.16175 (10)	0.0312 (5)	
C24	0.4888 (5)	0.2490 (2)	0.18037 (15)	0.0498 (8)	
H24A	0.4300	0.2477	0.2207	0.060*	
C25	0.4481 (5)	0.3018 (2)	0.13331 (17)	0.0565 (9)	
H25A	0.3575	0.3445	0.1341	0.068*	
C26	0.5728 (6)	0.3243 (2)	0.02215 (17)	0.0655 (11)	
H26A	0.5991	0.2830	−0.0110	0.098*	
H26B	0.6716	0.3660	0.0232	0.098*	
H26C	0.4553	0.3519	0.0130	0.098*	
N31	0.9945 (3)	−0.02052 (14)	0.06370 (11)	0.0338 (5)	
C32	0.8538 (4)	−0.00141 (17)	0.10051 (13)	0.0337 (6)	
H32A	0.7312	−0.0201	0.0934	0.040*	
N33	0.9037 (3)	0.04690 (13)	0.14854 (10)	0.0307 (5)	
C34	1.0903 (4)	0.05768 (18)	0.14078 (14)	0.0370 (7)	
H34A	1.1679	0.0894	0.1680	0.044*	
C35	1.1459 (4)	0.01614 (19)	0.08845 (14)	0.0404 (7)	
H35A	1.2678	0.0132	0.0722	0.048*	
C36	0.9860 (5)	−0.0720 (2)	0.00624 (14)	0.0504 (9)	
H36A	0.8635	−0.0974	0.0027	0.076*	
H36B	1.0795	−0.1161	0.0087	0.076*	
H36C	1.0094	−0.0371	−0.0311	0.076*	
N41	0.3159 (3)	−0.03724 (13)	0.27391 (11)	0.0325 (5)	
C42	0.4558 (4)	−0.02174 (16)	0.23532 (13)	0.0343 (6)	
H42A	0.4859	−0.0540	0.1989	0.041*	
N43	0.5474 (3)	0.04449 (13)	0.25430 (10)	0.0305 (5)	
C44	0.4575 (4)	0.07224 (17)	0.30800 (13)	0.0365 (7)	
H44A	0.4918	0.1196	0.3325	0.044*	
C45	0.3142 (4)	0.02213 (17)	0.32034 (14)	0.0358 (7)	
H45A	0.2290	0.0269	0.3544	0.043*	
C46	0.1797 (5)	−0.1034 (2)	0.26619 (17)	0.0515 (8)	
H46A	0.2137	−0.1385	0.2299	0.077*	
H46B	0.0582	−0.0786	0.2587	0.077*	
H46C	0.1757	−0.1375	0.3048	0.077*	
B1	0.5668 (5)	0.29807 (19)	0.39058 (15)	0.0315 (7)	
F11	0.4370 (3)	0.35592 (14)	0.40508 (12)	0.0798 (7)	
F12	0.7350 (3)	0.32732 (13)	0.40642 (11)	0.0793 (6)	
F13	0.5591 (3)	0.28377 (13)	0.32566 (8)	0.0665 (6)	
F14	0.5315 (4)	0.22546 (12)	0.42041 (9)	0.0711 (7)	
B2	0.4462 (5)	0.5760 (2)	0.55754 (18)	0.0425 (8)	
F21	0.3564 (6)	0.5904 (2)	0.50432 (16)	0.1491 (16)	
F22	0.3308 (4)	0.54048 (14)	0.60095 (16)	0.1142 (12)	
F23	0.5926 (4)	0.52453 (16)	0.54884 (12)	0.0815 (7)	
F24	0.5047 (4)	0.65008 (14)	0.58165 (13)	0.0866 (8)	

### Atomic displacement parameters (Å^2^)

**Table:**

	*U*^11^	*U*^22^	*U*^33^	*U*^12^	*U*^13^	*U*^23^
Zn1	0.02814 (15)	0.02826 (14)	0.02736 (14)	−0.00132 (17)	0.00093 (17)	−0.00254 (11)
N11	0.0360 (13)	0.0347 (12)	0.0339 (12)	0.0090 (11)	−0.0098 (11)	−0.0097 (11)
C12	0.0330 (15)	0.0307 (13)	0.0362 (15)	0.0035 (13)	−0.0003 (12)	−0.0038 (12)
N13	0.0317 (12)	0.0280 (11)	0.0281 (13)	0.0022 (10)	−0.0018 (9)	−0.0032 (9)
C14	0.0300 (15)	0.0285 (13)	0.0407 (16)	−0.0015 (12)	−0.0010 (12)	−0.0005 (12)
C15	0.0318 (15)	0.0311 (14)	0.0510 (18)	0.0002 (13)	−0.0101 (14)	−0.0039 (14)
C16	0.065 (2)	0.057 (2)	0.0345 (17)	0.0133 (19)	−0.0171 (16)	−0.0096 (15)
N21	0.0469 (15)	0.0389 (14)	0.0362 (14)	−0.0066 (13)	−0.0142 (12)	0.0034 (11)
C22	0.0433 (16)	0.0365 (15)	0.0369 (16)	0.0026 (14)	0.0046 (14)	0.0037 (13)
N23	0.0314 (13)	0.0344 (12)	0.0277 (12)	0.0015 (11)	0.0004 (10)	0.0000 (10)
C24	0.0482 (19)	0.066 (2)	0.0353 (16)	0.0203 (18)	0.0006 (15)	−0.0016 (16)
C25	0.059 (2)	0.058 (2)	0.053 (2)	0.0250 (18)	−0.0099 (18)	−0.0019 (18)
C26	0.080 (3)	0.065 (2)	0.052 (2)	−0.012 (2)	−0.019 (2)	0.0273 (19)
N31	0.0410 (14)	0.0300 (11)	0.0305 (12)	0.0088 (11)	−0.0007 (11)	−0.0019 (10)
C32	0.0363 (15)	0.0315 (14)	0.0333 (15)	0.0013 (13)	0.0002 (12)	−0.0043 (12)
N33	0.0332 (13)	0.0298 (11)	0.0292 (12)	0.0011 (10)	0.0003 (10)	−0.0035 (10)
C34	0.0325 (16)	0.0382 (16)	0.0402 (16)	−0.0008 (13)	−0.0006 (13)	−0.0061 (13)
C35	0.0348 (16)	0.0446 (17)	0.0418 (17)	0.0084 (15)	0.0033 (14)	−0.0020 (14)
C36	0.068 (2)	0.0482 (18)	0.0348 (16)	0.0147 (17)	−0.0050 (16)	−0.0144 (14)
N41	0.0318 (13)	0.0259 (11)	0.0398 (14)	−0.0037 (9)	0.0071 (9)	0.0009 (10)
C42	0.0392 (17)	0.0290 (14)	0.0347 (14)	−0.0028 (13)	0.0059 (13)	−0.0036 (12)
N43	0.0321 (13)	0.0270 (11)	0.0323 (12)	−0.0026 (11)	0.0022 (10)	−0.0018 (9)
C44	0.0406 (17)	0.0319 (14)	0.0371 (16)	−0.0011 (13)	0.0063 (13)	−0.0104 (12)
C45	0.0328 (16)	0.0367 (15)	0.0380 (15)	0.0040 (12)	0.0080 (12)	−0.0025 (13)
C46	0.0502 (19)	0.0400 (17)	0.064 (2)	−0.0149 (15)	0.0116 (16)	−0.0043 (16)
B1	0.0349 (18)	0.0275 (15)	0.0321 (17)	0.0002 (15)	−0.0037 (14)	0.0007 (13)
F11	0.0721 (15)	0.0666 (13)	0.1008 (18)	0.0343 (13)	−0.0033 (13)	−0.0135 (13)
F12	0.0497 (12)	0.0719 (12)	0.1162 (18)	−0.0095 (13)	−0.0260 (15)	−0.0203 (12)
F13	0.0923 (16)	0.0784 (14)	0.0290 (10)	−0.0241 (13)	0.0019 (10)	0.0042 (9)
F14	0.129 (2)	0.0406 (10)	0.0436 (11)	−0.0129 (12)	0.0012 (12)	0.0122 (9)
B2	0.037 (2)	0.0398 (19)	0.050 (2)	−0.0009 (17)	−0.0064 (17)	0.0080 (16)
F21	0.208 (4)	0.119 (2)	0.120 (3)	0.002 (2)	−0.118 (3)	0.021 (2)
F22	0.105 (2)	0.0549 (14)	0.182 (3)	0.0142 (14)	0.076 (2)	0.0322 (17)
F23	0.0736 (16)	0.0789 (15)	0.0921 (18)	0.0272 (14)	0.0142 (13)	0.0017 (13)
F24	0.0853 (17)	0.0659 (14)	0.109 (2)	−0.0175 (14)	0.0057 (16)	−0.0223 (14)

### Geometric parameters (Å, °)

**Table:**

Zn1—N43	1.980 (2)	N31—C36	1.465 (4)
Zn1—N13	1.982 (2)	C32—N33	1.324 (3)
Zn1—N33	1.983 (2)	C32—H32A	0.9500
Zn1—N23	1.991 (2)	N33—C34	1.375 (4)
N11—C12	1.330 (4)	C34—C35	1.348 (4)
N11—C15	1.359 (4)	C34—H34A	0.9500
N11—C16	1.473 (4)	C35—H35A	0.9500
C12—N13	1.322 (3)	C36—H36A	0.9800
C12—H12A	0.9500	C36—H36B	0.9800
N13—C14	1.366 (3)	C36—H36C	0.9800
C14—C15	1.347 (4)	N41—C42	1.324 (4)
C14—H14A	0.9500	N41—C45	1.364 (4)
C15—H15A	0.9500	N41—C46	1.459 (4)
C16—H16A	0.9800	C42—N43	1.314 (3)
C16—H16B	0.9800	C42—H42A	0.9500
C16—H16C	0.9800	N43—C44	1.378 (3)
N21—C22	1.320 (4)	C44—C45	1.339 (4)
N21—C25	1.362 (4)	C44—H44A	0.9500
N21—C26	1.463 (4)	C45—H45A	0.9500
C22—N23	1.317 (4)	C46—H46A	0.9800
C22—H22A	0.9500	C46—H46B	0.9800
N23—C24	1.357 (4)	C46—H46C	0.9800
C24—C25	1.335 (5)	B1—F14	1.346 (4)
C24—H24A	0.9500	B1—F12	1.349 (4)
C25—H25A	0.9500	B1—F11	1.356 (4)
C26—H26A	0.9800	B1—F13	1.386 (4)
C26—H26B	0.9800	B2—F21	1.316 (4)
C26—H26C	0.9800	B2—F23	1.357 (4)
N31—C32	1.318 (4)	B2—F24	1.359 (4)
N31—C35	1.350 (4)	B2—F22	1.364 (4)
			
N43—Zn1—N13	108.27 (9)	N31—C32—N33	111.9 (3)
N43—Zn1—N33	118.81 (9)	N31—C32—H32A	124.1
N13—Zn1—N33	107.97 (9)	N33—C32—H32A	124.1
N43—Zn1—N23	106.57 (9)	C32—N33—C34	104.6 (2)
N13—Zn1—N23	110.09 (9)	C32—N33—Zn1	129.2 (2)
N33—Zn1—N23	104.93 (9)	C34—N33—Zn1	125.57 (19)
C12—N11—C15	106.9 (2)	C35—C34—N33	109.2 (3)
C12—N11—C16	126.4 (3)	C35—C34—H34A	125.4
C15—N11—C16	126.6 (3)	N33—C34—H34A	125.4
N13—C12—N11	111.3 (3)	C34—C35—N31	106.7 (3)
N13—C12—H12A	124.3	C34—C35—H35A	126.7
N11—C12—H12A	124.3	N31—C35—H35A	126.7
C12—N13—C14	105.7 (2)	N31—C36—H36A	109.5
C12—N13—Zn1	127.5 (2)	N31—C36—H36B	109.5
C14—N13—Zn1	126.43 (18)	H36A—C36—H36B	109.5
C15—C14—N13	108.9 (2)	N31—C36—H36C	109.5
C15—C14—H14A	125.6	H36A—C36—H36C	109.5
N13—C14—H14A	125.6	H36B—C36—H36C	109.5
C14—C15—N11	107.1 (2)	C42—N41—C45	108.4 (2)
C14—C15—H15A	126.4	C42—N41—C46	126.0 (2)
N11—C15—H15A	126.4	C45—N41—C46	125.5 (2)
N11—C16—H16A	109.5	N43—C42—N41	110.7 (2)
N11—C16—H16B	109.5	N43—C42—H42A	124.7
H16A—C16—H16B	109.5	N41—C42—H42A	124.7
N11—C16—H16C	109.5	C42—N43—C44	105.7 (2)
H16A—C16—H16C	109.5	C42—N43—Zn1	129.52 (19)
H16B—C16—H16C	109.5	C44—N43—Zn1	124.14 (18)
C22—N21—C25	106.6 (3)	C45—C44—N43	109.5 (2)
C22—N21—C26	126.0 (3)	C45—C44—H44A	125.3
C25—N21—C26	127.4 (3)	N43—C44—H44A	125.3
N23—C22—N21	111.8 (3)	C44—C45—N41	105.8 (2)
N23—C22—H22A	124.1	C44—C45—H45A	127.1
N21—C22—H22A	124.1	N41—C45—H45A	127.1
C22—N23—C24	105.3 (2)	N41—C46—H46A	109.5
C22—N23—Zn1	126.5 (2)	N41—C46—H46B	109.5
C24—N23—Zn1	128.2 (2)	H46A—C46—H46B	109.5
C25—C24—N23	109.6 (3)	N41—C46—H46C	109.5
C25—C24—H24A	125.2	H46A—C46—H46C	109.5
N23—C24—H24A	125.2	H46B—C46—H46C	109.5
C24—C25—N21	106.8 (3)	F14—B1—F12	110.9 (3)
C24—C25—H25A	126.6	F14—B1—F11	110.7 (3)
N21—C25—H25A	126.6	F12—B1—F11	109.6 (3)
N21—C26—H26A	109.5	F14—B1—F13	108.0 (2)
N21—C26—H26B	109.5	F12—B1—F13	109.7 (3)
H26A—C26—H26B	109.5	F11—B1—F13	107.9 (3)
N21—C26—H26C	109.5	F21—B2—F23	112.3 (4)
H26A—C26—H26C	109.5	F21—B2—F24	108.5 (3)
H26B—C26—H26C	109.5	F23—B2—F24	109.7 (3)
C32—N31—C35	107.6 (2)	F21—B2—F22	109.8 (4)
C32—N31—C36	125.7 (3)	F23—B2—F22	108.5 (3)
C35—N31—C36	126.7 (3)	F24—B2—F22	107.8 (3)
